# Induced polyploidy deeply influences reproductive life cycles, related phytochemical features, and phytohormonal activities in blackberry species

**DOI:** 10.3389/fpls.2022.938284

**Published:** 2022-08-12

**Authors:** Nasrin Sabooni, Ali Gharaghani

**Affiliations:** Department of Horticultural Science, School of Agriculture, Shiraz University, Shiraz, Iran

**Keywords:** phytohormones, polyploidy, flowering, fruiting, nectar, breeding

## Abstract

In some cases, polyploidy is an important phenomenon in the evolution of fruit crops. Polyploidy can be used in fruit breeding programs to develop varieties with higher yields and better fruit quality, as well as better adaptation to adverse environmental conditions. In this study, three wild species of blackberry were subjected to different degrees of induced polyploidy, and the effects of which were evaluated on morphological, physiological, and phytohormonal traits. With the aim of gaining a deep insight into the generative phase of plant growth and development, different levels of induced polyploidy were evaluated on the three blackberry species, i.e., *Rubus persicus* Bioss. (*2x, 4x*, and *8x*), *R. caesius* L. (*2x* and *4x*), and *R. hirtus* Schreb. *(2x* and *4x*). The results showed that the polyploid plants performed significantly better than their diploid counterparts in terms of morphological traits such as flower count per spike and berry weight, as well as biochemical traits such as total soluble solids in the leaves. Induced polyploidy increased berry weight and drupe count per fruit. Microscopic examinations revealed a smaller number of viable pollen in the polyploids, compared to the diploids. Electron microscopy showed that the octaploid *R. persicus* had larger conical cells on the flower surface, compared to the diploid *R. persicus*. Correlation analysis showed that the ratio of indoleacetic acid to jasmonic acid changed synergistically with the total soluble solids in the leaves during the fruit set. The ploidy level correlated significantly with the number of pistils, leaf green index, total soluble solids in the leaves, and glucose content in floral nectar. Overall, induced polyploidy allowed *Rubus* to develop advantageous traits that can benefit future breeding programs and expand reproductive research in blackberries.

## Graphical Abstract

Created with BioRender.com. 
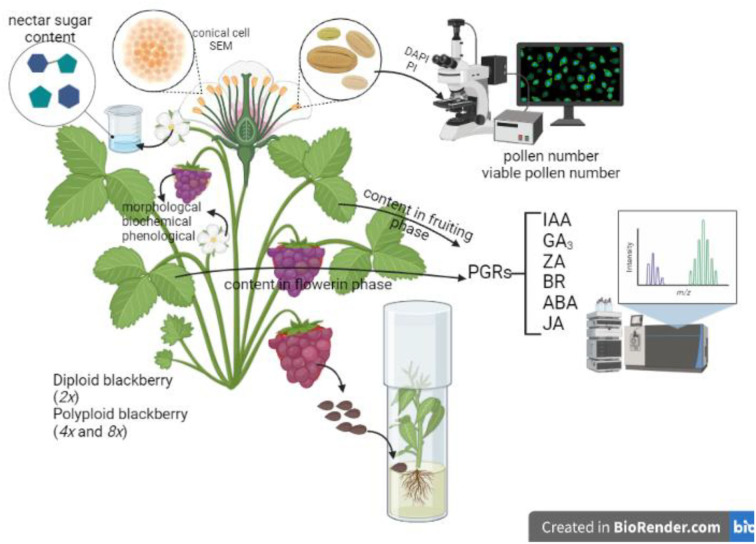

## Introduction

Polyploidy is a common phenomenon in angiosperms that have played an important role in plant evolution. Polyploidy is estimated to have occurred in 40–70% of all plant species, implying that hybridization is a widespread method of promoting adaptation in plants (Mason, 2017). Most organisms have genomes with a large degree of gene redundancy, which usually results from the duplication of the whole genome (Martin et al., 2019). Molecular evaluation of plant genomes has shown that recurrent polyploidy in the plant kingdom is the rule, rather than the exception (Peer et al., 2017). Genetic studies confirmed that autopolyploidy is far more common than not, while uncovering the underlying genetic causes for the success of these species (Soltis et al., 2004; Mason, 2017). Flowering plants with *n* = 14 or more chromosomes have been considered as polyploid (Dar and Rehman, 2017). Polyploids generally differ from diploids in their morphological and physicochemical characteristics. For example, the stomata in polyploid plants are generally much larger than those of their diploid counterparts. Accordingly, Masterson (1994) compared the size of stomata in ancient and contemporary species, so as to propose a valid method for evaluating the occurrence of polyploidy. As a result of the comparisons, it was estimated that 70 percent of all angiosperms had undergone one or more episodes of polyploidy. As a dynamic concept, the specifics of polyploidy make plants respond differently to the evolutionary constraints of polyploid formation (Jackson and Chen, 2010). Following gene duplication, both genetic and epigenetic pathways can influence gene expression. In some cases, it is possible that homologous loci become subfunctionalized, with one homolog in one organ and the other homolog in another organ being silenced (Barker et al., 2012; Jiang et al., 2013). During the polyploidization process, genomic shocks or genome interactions can occur, resulting in unique patterns of gene expression. Transposons and retrotransposons can be transcriptionally and transpositionally activated as a consequence of polyploidy (Soltis et al., 2004; Jackson and Chen, 2010).

In recent decades, plant breeders have considered polyploidy as an efficient, rapid breeding technique to develop healthy cultivars (Braynen et al., 2017). Polyploidy-based breedingprograms combine the advantages of heterosis and apomixes, thereby offering a promising approach to plant breeding in the future (Comai, 2005). Research on induced polyploidy has contributed to a more specific understanding of polyploid evolution and is expected to assist in plant breeding (Wang et al., 2022). In this context, tetraploids can be used as parental material in breeding programs to develop seedless triploid varieties with improved quality and better environmental adaptation in many crops, such as grapes, mandarins, etc. (Bahadur et al., 2015). Polyploidy is a crucial step in vegetative and generative growth, as it affects plant development. Thus, the effects of polyploidy deserve to be evaluated on fruit set and seed production in breeding. In autotetraploid plants, changes in the regulatory system of flower development are limited (Braynen et al., 2021). Compared to diploid plants, tetraploids are sometimes characterized by larger fruits, lack of seeds, higher plant production, and changes in the metabolism of secondary metabolites (Bertea et al., 2005; Ravandi et al., 2015). For example, autotetraploid watermelons reportedly produced seedless fruits (Compton et al., 1996; Wehner, 2008). Tetraploid *Acorus calamus* showed a significant increase (95%) in anticancer chemical compounds, compared to the amount produced by diploid plants of the same species (Bertea et al., 2005). Similarly, tetraploid *Cichorium intybus* reportedly showed higher levels of phenolic compounds and chlorogenic acid (Ravandi et al., 2015).

Blackberries are considered superfruits because of their high nutritional value. Antioxidants, anthocyanins, phenolic substances and flavonoids in blackberry fruits may reduce the incidence of obesity, coronary heart disease, degenerative diseases, and various forms of cancer, according to epidemiological and clinical research (Lee, 2018). The literature on the genetic resources of this plant indicates extensive research on breeding and horticultural use. Natural polyploidy has occurred in the genus *Rubus*.

Apomixis is also very common in the Rosaceae family, which includes *Rubus* species. Apomixis, hybridization, and polyploidy have had significant taxonomic impacts on the Rosaceae family, particularly in *Rubus* spp. (Dickinson et al., 2007), *Malus x domestica* (Xue et al., 2015; Hias et al., 2017), *Pyrus pyriflora* (Kadota and Niimi, 2002), *Pyrus communis* (Sun et al., 2009), *Prunus laurocerasus* (Contreras and Meneghelli, 2016), and strawberry (Dickinson et al., 2007).

Polyploidy is an important phenomenon in the evolution of fruit crops. Inducing it artificially can assist in fruit breeding programs to develop varieties with a higher yield, better fruit quality, and stronger mechanisms for adaptation to adverse environmental conditions. In this study, different levels of induced polyploidy were evaluated on three wild blackberry species, i.e., *Rubus persicus* Bioss. (*2x, 4x*, and *8x*), *R. caesius* L. (*2x* and *4x*), and *R. sanctus* Schreb. (*2x* and *4x*). The effects of induced polyploidy were measured in terms of morphological, physiological, and biochemical traits, which helped with a deep insight into the generative phase of plant growth and development. The main objective was to examine the effects of increased ploidy level on reproduction, generative organs, and fruit characteristics of the different blackberry species. Other aspects of this study involved evaluating how the different ploidy levels caused variations in phytochemicals and phytohormones in the reproductive life cycle of the plants. To the best of our knowledge, this study is the first to examine artificially-induced octaploidy in blackberry.

## Materials and methods

### Plant materials

Autopolyploidy was artificially induced in plants by oryzalin or colchicine using an *in vitro* protocol (Sabooni et al., 2022). The plants were of three species, with different ploidy levels, *R. persicus* Bioss. (*2x, 4x*, and *8x*), *R. caesius* L. (*2x* and *4x*), and *R. sanctus* Schreb. (*2x* and *4x*). Flow cytometry analysis was employed to validate the somatic ploidy level (Sabooni et al., 2022). The plants were grown in a greenhouse with a 16/8-h light/dark photoperiod and 25/18 ± 2°C (day/night) temperature. All plant genotypes bloomed in the second growing season, after providing chilling requirements in the controlled greenhouse. The morphological traits were measured for two growing seasons (2020 and 2021), but the phytochemical and phytohormonal assessments were made only in the second growing season (2021). Fully expanded leaves (during the blooming stage) and ripened fruits were harvested, placed promptly in liquid nitrogen, and then stored at −80°C for further analysis. Meanwhile, morphological characterization was performed on fresh leaves, flowers, and fruits. The samples were collected in three replicates.

### Phenology

**Figure 1:**
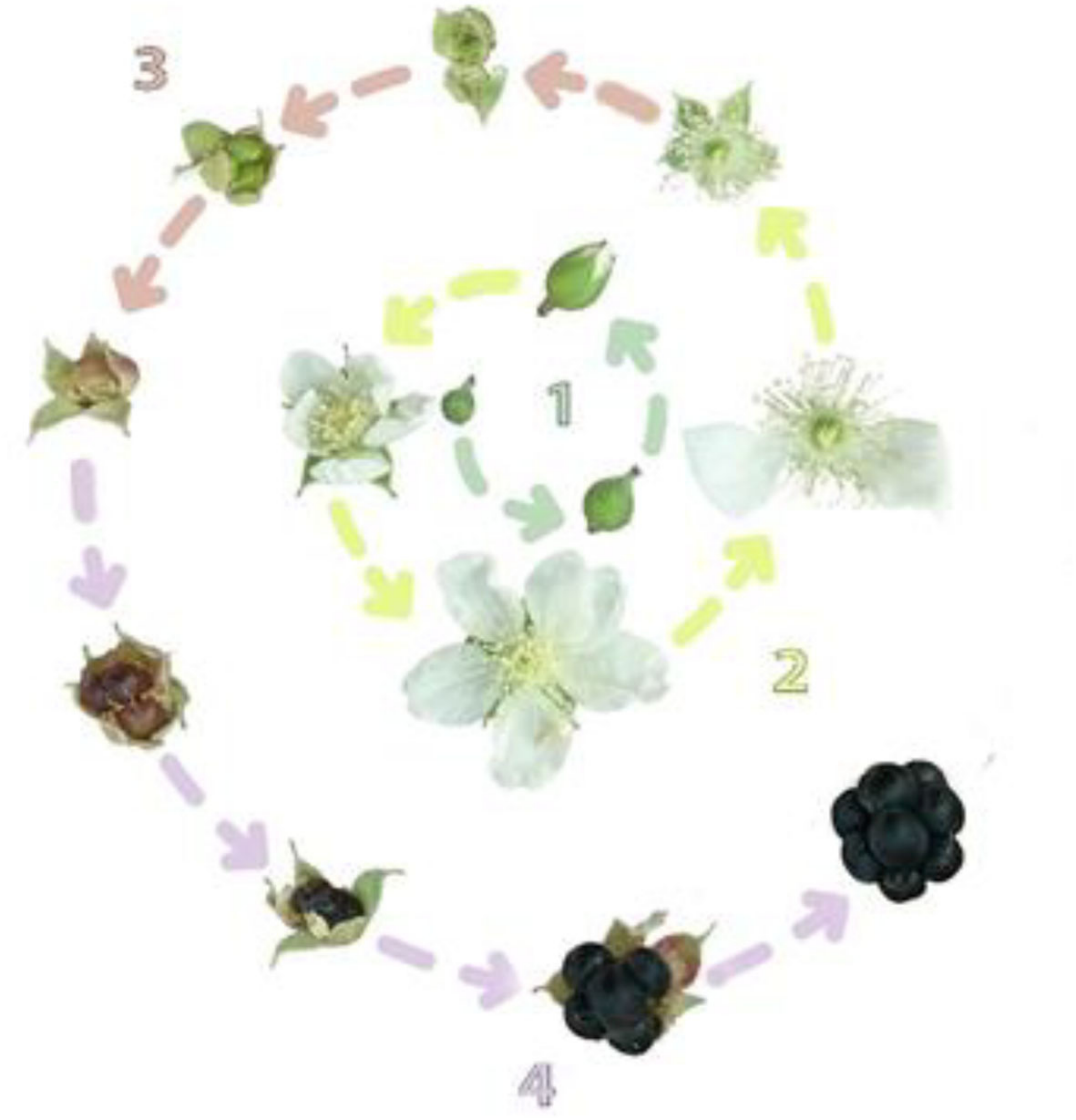
The life circle of blackberry bloom. 1: bud emergence to anthesis 2: anthesis to petal fall 3: fruit set duration 4: fruit growth duration 5: color development duration.

To record phenological data, the duration from the beginning of bud development to the end of fruit ripening was divided into five stages (Figure 1): (1) flower bud development (bud break to anthesis), (2) flowering period (anthesis to petal fall), (3) fruit set, (4) fruit growth, (5) fruit color development.

### Morphological traits

The number of clusters per plant, number of flowers per cluster, number of stamens and pistils per flower, number of fruits per cluster, and number of drupelets per fruit were counted on replicated diploid and polyploidy plants of each species. A digital scale (0.001 g) was used for measuring the fruit weight. A vernier caliper was used for measuring the flowers, fruits, and drupelets in diameter.

### Electron microscopy

Scanning electron microscopy (SEM) was conducted according to the procedures described by Ghanbari et al. (2019). For this purpose, mature petals were collected from mother (diploid) and polyploid plants, washed with distilled water, and dried carefully with soft tissue. Dental wax was used for fabricating replicas from the petals. Apoxy glue and a hardener were employed to fill the negative duplicate (ratio 2:1). The SEM TESCAN-Vega 3 (Brno, Czech Republic) instrument was used for imaging the positive replica.

### Biochemical assessments

The total soluble solids of fruits (TSS_F_) were measured by using a digital refractometer (Milwaukee MA871, Hungary). The amount of ascorbic acid (vitamin C) in the fruit juice was determined according to the 2, 6-dichlorophenol indophenol technique (AOAC, 2000). The Folin-Ciocalteu colorimetric technique was employed to determine the total phenolic content (TPC) (Du et al., 2009). For this purpose, 700 μl of fruit juice was combined with 900 μl of 2% sodium carbonate and kept at room temperature for a few minutes. Then, 180 μl of 50% folin was added, and the samples were kept at room temperature for 30 mins. A microplate spectrophotometer (Epoch Biotech, Germany) was used for measuring the absorbance of mixtures at 750 nm wavelength. The total phenolic content was expressed as mg of GAE l^−1^.

The pH differential spectrophotometry technique was used for determining anthocyanin concentrations in the fruits (Habibi et al., 2020). Two buffer schemes were employed, i.e., pH 1.0, along with potassium chloride buffer (25 mM), and pH 4.5, along with sodium acetate buffer (400 mM). A microplate spectrophotometer (Epoch Biotech, Germany) was used for measuring the absorbance at 510 and 700 nm. Total anthocyanin (mg l^−1^) was expressed as cyanidin-3-glucoside equivalents using Equations 1 and 2:


(1) $$\begin{array}{l} \text{A }=\;(\text{A}\lambda \text{vis}-\text{max}-\text{A}\lambda 700\text{ nm})\text{ pH 1}.0\\ \;-(\text{A}\lambda \text{vis}-\text{max}-\text{A}\lambda 700\text{ nm})\text{ pH 4}.\text{5} \end{array}$$

(2) (A×MW×DF×1000)/(ε×l)

A: sample absorption rate, A λvis-max: maximum absorption, MW: molecular weight, cyanidin-3-glucoside (gmol^−1^), DF: dilution factor, ε: 26,900, molar absorption of cyanidin-3-glucoside, and L: well diameter (cm).

The DPPH free radical scavenging technique was used for assessing antioxidant activity in the fruits (Brand-Williams et al., 1995). Accordingly, 100 μl fruit juice was combined with 1 ml DPPH (0.1 mM) and 1 ml Tris-HCl (pH = 7.5) buffer. The mixture was stored at room temperature for 30 mins. The absorbance was measured at 517 nm using a microplate spectrophotometer (Model Epoch Biotech, Germany). Finally, the following formula was used for determining antioxidant activity:


Antioxidantactivity(%)=(1-Asample/Asample) ∗ 100

Total soluble solids in the leaves (TSS_L_) were calculated using the technique described by Patade et al. (2012). The closest leaf to a flower cluster was picked; 100 mg of its tissue was crushed and combined with 10 ml of an ethanol/distilled water solution (8:2 v/v). Then, it was allowed to settle at room temperature for 30 mins. The mixture was centrifuged at 4°C for 15 mins at 12,000 rpm. The supernatant was then combined with 3 ml of Anthrone reagent and incubated in a water bath at 100°C for 15 mins before being chilled on ice. Finally, the spectral absorbance was determined at 620 nm using a microplate spectrophotometer (Model Epoch Biotech, Germany). The standard was glucose, and the results were expressed as mg/g of dry weight.

A portable chlorophyll meter, the SPAD-502 (Konica Minolta Inc., Tokyo, Japan), was used for estimating the chlorophyll content. During the flowering stage, the first fully-expanded leaf (from the spike) of each plant was examined. Each leaf was examined three times, each time at a different location. The average of three observations was used for calculating the chlorophyll concentration of each leaf.

### Pollen, seed characterization, and viability

Pollen were collected from five blossoming flowers of mother and polyploid plants. The samples created a temporary slide to be examined under a fluorescence microscope Lionheart FX (Biotek, USA). For staining and counting the average number of pollen, DAPI fluorescent dye was used, while a sensitive dye for PI fluorescent light was used for counting viable pollen.

Twenty seeds were extracted from the ripe fruits of each replicate. After stratification and scarification with a nail file, the seeds were disinfected with 70% alcohol and 15% chlorax. These seeds were then cultivated *in vitro* on an MS medium. Germinated seeds were counted for 3 months.

### Profiling nectar sugars

Five fresh burgeoned flowers were collected from each plant. Each flower was immersed and shaken in distilled water (2 ml) for 30 s, without the petiole entering the water. The samples were then concentrated and the final product was dissolved in HPLC grade methanol. The extract was filtered using a 0.22 μm syringe filter, and 10 μl of it was fed into the HPLC system (Knauer HPLC 1000 bar). The elution method involved using 0.1% phosphoric acid that flowed isocratically at a rate of 500 μl min^−1^. Sugars were eluted on a Eurokat column (Eurokat H, 10 μm, Column 300 × 8 mm—KNAUER—Germany) and detected at 210 nm using a refractive index detector. Standard curves of pure sucrose (suc), glucose (glu), and fructose (fru) were used for quantifying the sugars.

### Profiling phytohormones

A method by Pan et al. (2010) was used for extracting leaf hormones, which enabled the profiling of phytohormones. For this purpose, the nearest leaf to a spike was used and 50 mg of frozen leave tissue was placed in a 2 ml tube filled with 500 μl of extraction solvent (2/1/0.002: isopropanol/H_2_O/concentrated HCl: v/v/v). After 30 mins of shaking at 4°C, the tubes were filled with 1 mL dichloromethane and shaken again at 4°C for 30 mins. The samples were placed in a chilled micro-centrifuge at 4°C and centrifuged at 13,000 g for 5 mins, with the bottom phase being transferred to a screw-cap vial to concentrate the mixture. Finally, the samples were dissolved in 100 μl methanol. After injecting standard solutions, calibration curves were demonstrated for each plant growth regulator.

The liquid chromatography mass spectrometry (LC-MS) analysis was carried out on an ACQUITY UPLC H-lass System device with an ACQUITY QDa Mass Detector (Waters Corporation, Milford, MA, USA), while using the analytical column C18 (2.1 50 mm, 1.7 m; Waters Corporation). The flow rate was 500 μl min^−1^, and the 5,000 μl of injection volume was eluted by 30% (v/v) with an isocratic mixture of HPLC-grade water for all phytohormones. Different concentrations of standard solutions (0.002–2,000 μg ml^−1^) were generated and utilized to plot the standard curves. The information was expressed as micrograms per kilogram of fresh weight (μg kg^−1^ fw^−1^) of indole acetic acid (IAA), zeatin (ZA), gibberellic acid (GA_3_), abscisic acid (ABA), jasmonic acid (JA), and brasinoestroid (BR).

### Statistical analysis and experimental design

All statistical analyses were performed in the R software (version4.0.5). Morphological data which had been gathered for 2 years were compared to determine the significance of each year. Then, the average data of the 2 years were used because the effect of the year and year×genotype were not significant (*p*< 0.05) for almost all of the measured traits. A completely randomized design (CRD) with three replicates was used for analyzing the effects of ploidy level. Mean values were compared by using the LSD test. Minitab software (version16) was used for evaluating the relationship between ploidy level and other parameters using principal component analysis (PCA). To examine the nature and degree of the relationships between all measured factors, Pearson's correlation coefficient was computed by R. The heat map was also produced using corrplot packages in the blooming and flowering stages.

## Results

### Phenological parameters

Almost all plant genotypes flowered in the second growing season after meeting cold requirements in the controlled environment of the greenhouse. Flowering occurred regardless of species or ploidy level. Looking at the effects of induced polyploidy on flowering and fruiting phenology, the three species responded differently in this regard (Figure 1; Table 1). While increased polyploidy shortened the duration of flower bud development (bud break to anthesis) in *R. persicus* and *R. caesius*, the opposite trend was observed in *R. sanctus*. The maximum (30.33 days) and minimum (11.33 days) number of days required for anthesis (bud break to anthesis) were observed in diploid *R. caesius* and *R. sanctus*, respectively. Polyploidy generally prolonged flowering time (anthesis to petal fall), but this effect was not statistically significant in *R. persicus* and *R. caesius* between diploids and tetraploids. In addition, *R. persicus 2x* and *4x* had the shortest flowering time (4 days), while *R. persicus 8x* surprisingly had the longest flowering time (9 days), where petals remained on the flower base even until the fruit ripening. The duration of the fruit set decreased with increasing ploidy level, although this reduction was not statistically significant between diploids and tetraploids in *R. persicus* and *R. caesius*. The longest (15 days) and shortest (4 days) duration of the fruit set was observed in *R. persicus 2x* and *R. caesius 4x*, respectively. It is worth mentioning that the fruit set was shortened by 50% in octaploid *R. persicus* (7.33 days) compared to its diploid counterpart. Induced polyploidy prolonged the duration of the fruit set, with the exception of *R. persicus*, where an opposite trend was observed, especially in the octaploid genotype. The longest and shortest duration of fruit growth was measured in diploid (21.66 days) and octaploid (7.66 days) *R. persicus*, respectively. The duration of fruit coloration decreased in response to an increase in ploidy level, except in *R. caesius*, where there were no differences between diploid and tetraploid plants. The duration of fruit coloration in diploid *R. persicus* (9.66 days) was about twice as long as its octaploid counterpart (5.33).

**Table 1:** Effect of ploidy level on phenological parameters of three *Rubus* species.

**Species**	**Flower development duration (bud emergence to anthesis) (days)**	**Flowering period (anthesis to petal fall) (days)**	**Fruit set duration (days)**	**Fruit growth duration (days)**	**Fruit color development duration (days)**
*R. persicus 2x*	24.00^a^	4.00^b^	15.00^a^	21.66^a^	9.66^a^
*R. persicus 4x*	17.33^b^	4.00^b^	14.33^a^	20.66^a^	8.00^a^
*R. persicus 8x*	17.00^b^	^**+**^9.00^a^	7.33^b^	7.66^b^	5.33^b^
	*	*	*	*	*
*R. caesius 2x*	30.33^a^	5.00	5.00	12.00^b^	5.00
*R. caesius 4x*	23.66^b^	4.00	4.00	15.33^a^	5.33
	*	ns	ns	*	ns
*R. sanctus 2x*	11.33^b^	5.33^b^	9.00^a^	9.66^b^	12.00^a^
*R. sanctus 4x*	16.33^a^	7.33^a^	4.66^b^	13.00^a^	8.00^b^
	*	*	*	*	*

^+^R. persicus 8x, did not abscise its petal up to fruit ripening, thus the first signs of fruit set considered as the end of flowering period in this genotype.

In each column and species, means followed by the same letters are not significantly different according to LSD test.

The “^*^” was significant at P ≤ 0.05 and the “ns” letters were not significant.

### Morpho-chemical characteristics

**Figure 2:**
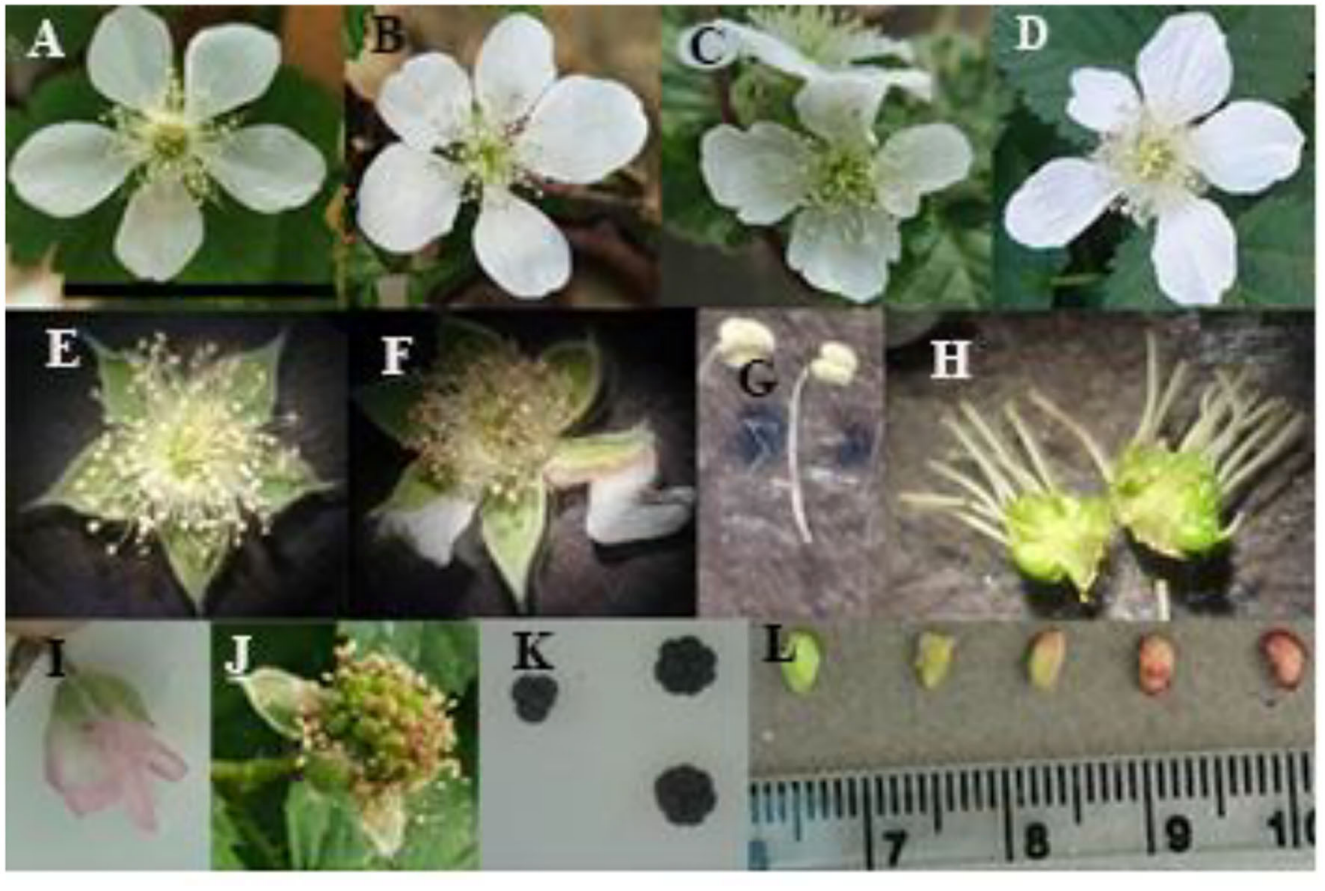
Morphological characteristics of flower, fruit, and seed in diploid and polyploid blackberries. **(A)** Diploid flower, **(B)** tetraploid flower, **(C,D)** octaploid flower, **(E)** normal stamen, anther, and sepal, **(F)** browned anther, abnormal sepal in tetraploid flower, **(G)** diploid (right) and polyploid (left) stamen, **(H)** diploid (left) and octaploid (right) ovary and pistil, **(I)** change in color of petal after pollination in polyploid plants, **(J)** fruit set, **(K)** fruit size and shape in diploid (left) and octaploid (right), **(L)** different stages of seed growth (the scale bar showed 25 mm).

**Figure 3:**
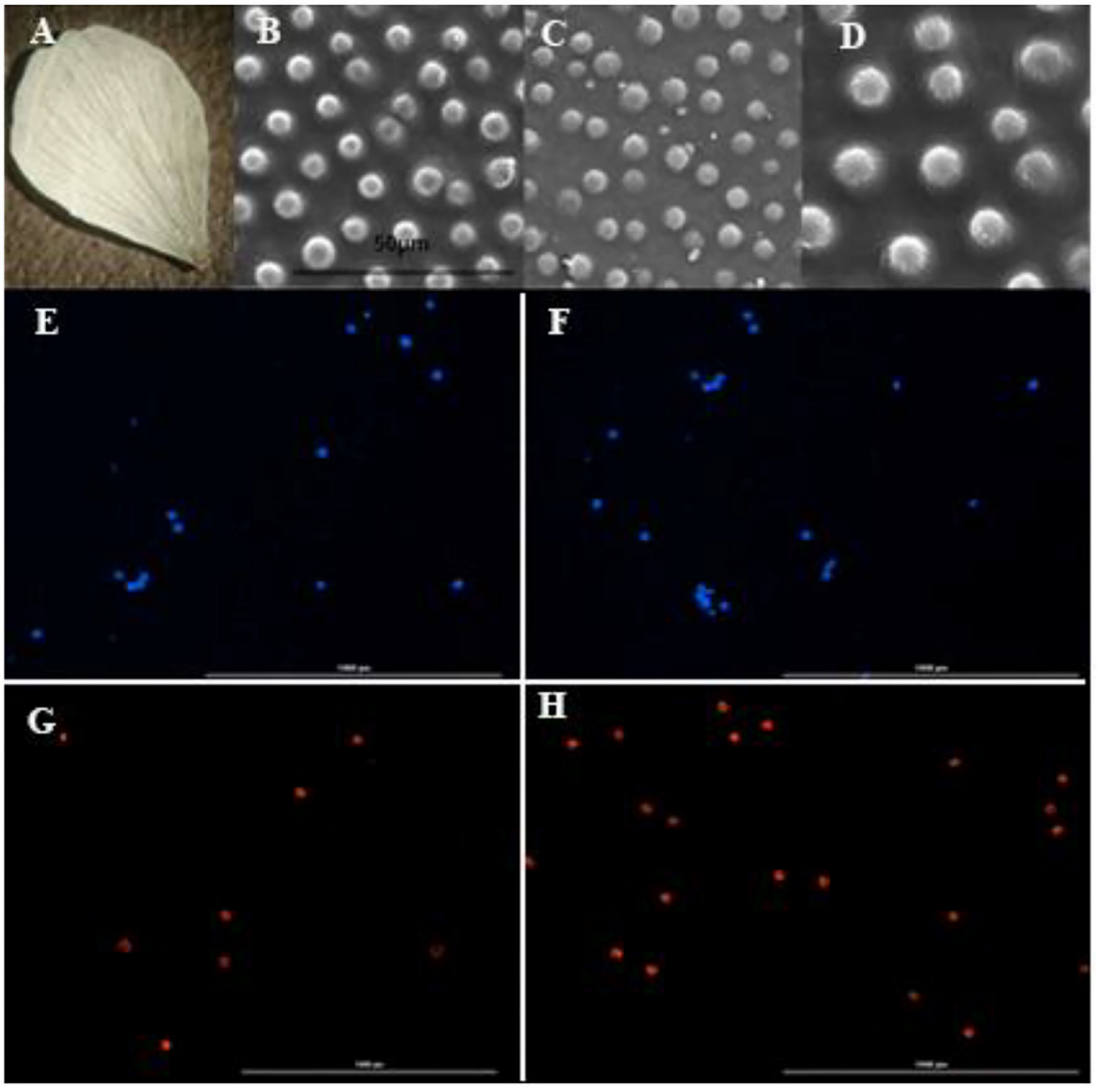
Electron microscopy of petal and fluorescent microscopy of pollens in diploid, tetraploid and octaploid plants of *Rubus persicus* blackberry. **(A)** Blackberry petal, **(B)** conical shape of diploid plants, **(C)** conical shape of tetraploid plants, **(D)** conical shape of octaploid plants, **(E)** pollen number of octaploid flower stained by DAPI, **(F)** pollen number of diploid flower stained by DAPI, **(G)** viable pollen number of octaploid flower stained by PI, **(H)** viable pollen number of diploid flower stained by PI. The scale bar in the pollen image showed 1,000 μm.

The results of measuring morpho-chemical characteristics on plants, leaves, flowers, and fruits are shown in Table 2 and in Figures 2, 3. The three species showed different responses to polyploidization, with regard to many of the morpho-chemical traits. Leaf green index and total soluble solids increased in all species when the ploidy level increased, except in the case of leaf green index in *R. caesius*, which was not affected by induced polyploidy. Among all species, the highest values of greenness index and TSSl were observed in *R. persicus 8x* (54.2 and 58.0 mg g^−1^DW^−1^, respectively). The number of clusters per plant decreased in *R. persicus* and *R. caesius*, while it increased in *R. sanctus* with increasing ploidy level, while the opposite trend was observed in the number of flowers per plant, which increased in all three species, except for the tetraploid *R. persicus*. Only in *R. persicus* did the size of the flower change, and the octaploid cytotypes had the largest flowers compared with the diploid and tetraploid cytotypes. Induced polyploidy increased the number of pistils in *R. persicus* (octaploid only) and *R. caesius*, whereas it did not affect this trait in *R. sanctus*. The number of stamens per flower increased only in *R. caesius* due to a higher degree of ploidy. A higher degree of ploidy resulted in a significantly lower number of pollen as well as a lower number of viable pollen in *R. persicus* and *R. caesius*, whereas in *R. sanctus*, the number of pollen increased and the number of viable pollen remained constant. However, the percentage of viable pollen was not significantly affected in any of the species.

**Table 2:** The mean comparison of leaf, flower, and fruit's morphological and biochemical characteristics between diploid and polyploid plants of three blackberry species.

**Plants**	**Greenness index**	**TSS_L_ (mg g^−1^dw^−1^)**	**Cluster/plant (no.)**	**Bloom diameter (mm)**	**Flower/cluster (no.)**	**Pistil (no.)**	**Stamen (no.)**	**Pollen (no.)**	**Viable pollen (no.)**	**Pollen viable %**	**Fruit/cluster (no.)**	**Drupelet/fruit (no.)**	**Fruit weight (g)**	**Fruit diameter (mm)**	**Drupelet diameter (mm)**	**TSS_F_ (°Brix)**	**Vit C (mgL^−1^)**	**Anthocyanin (mg L^−1^)**	**Antioxidant %**	**Phenol (mg of GAE L^−1^)**	**Seed germination %**
*R. persicus 2x*	36.5^b^	42.4^c^	10.0^a^	4.3^ab^	6.0^*ab^	16.0^ab^	107	190.0^a^	85.0^a^	44.7	2.4^b^	6.0^b^	0.6^b^	7.6^b^	5.0^b^	12	7.6	39.1^b^	60.3^b^	175.7^b^	0.60^a^
*R persicus 4x*	42.5^b^	49.5^b^	3.0^b^	4.0^b^	4.3^b^	12.3^b^	95	108.0^b^	51.0^b^	47.1	4.7^a^	10.6^ab^	0.9^b^	9.7^b^	4.9^b^	13.2	7.1	68.8^a^	72.2^ab^	155.4^c^	0.50^b^
*R. persicus 8x*	54.2^a^	58.0^a^	8.0^a^	5.5^a^	7.0^a^	22.6^a^	113.3	104.0^b^	43.0^b^	41.1	5.7^a^	18.6^a^	1.8^a^	13.5^a^	5.9^a^	10.8	8.8	41.0^b^	85.7^a^	222.7^a^	0.50^b^
	**	***	**	*	*	*	ns	***	***	ns	*	**	***	***	**	ns	ns	*	*	*	**
*R. caesius 2x*	24.9	43.4^b^	6.6	2.9	6.6^b^	10.0^b^	73.3^b^	30.0^a^	18.0^a^	59.7	5.5^a^	4.3	0.5^b^	10	4.8	19	9.2	64.6	74.7^a^	236.0^a^	0.50^a^
*R. caesius 4x*	24.2	48.8^a^	5	4.2	9.0^a^	16.3^a^	103.3^a^	9.3^b^	4.6^b^	50.5	3.2^b^	6.3	0.7^a^	10.9	5.3	22	8.0	88.7	64.9^b^	199.4^b^	0.50^a^
	ns	***	ns	ns	*	**	*	***	**	ns	*	ns	*	ns	ns	ns	ns	ns	*	***	*
*R. sanctus 2x*	25.6^b^	44.3^b^	2.3^b^	4.6	4.0^b^	15	113	39.0^b^	12	32	3.3	4.6	0.6^b^	11.3	5.6	16.4	7.1	29.1^b^	66.8^a^	233.3^a^	0.40^a^
*R. sanctus 4x*	28.1^a^	50.0^a^	8.3^a^	4.5	8.3^a^	15	106.6	60.0^a^	14	23.3	4.3	6.6	0.8^a^	19.2	5.7	16.4	8.6	62.2^a^	58.8^b^	174.7^b^	0.40^a^
	**	***	*	ns	*	ns	ns	**	ns	ns	ns	ns	*	ns	ns	ns	ns	***	**	**	*

^*^Means followed by the same letters in each species are not significantly different according to LSD test.

The “^***^” was significant at P ≤ 0.001, the “^**^” was significant at P ≤ 0.01, the “*” was significant at P ≤ 0.05 and the “ns” letters were not significant.

The number of fruits per cluster increased in *R. persicus*, decreased in *R. caesius*, and remained constant in *R. sanctus*, while the number of drupes per fruit increased only in *R. persicus*, especially in the octaploid cytotype. Berry size and weight increased significantly in the polyploid genotypes, compared with their diploid counterparts, with the octaploid *R. persicus* having the largest fruits (1.8 g compared with 0.6 g in its diploid counterpart). Induced polyploidy did not significantly affect the TSS and vitamin C content of the fruits in any of the species, while the anthocyanin content and antioxidant activity of the fruits were increased by polyploidization in almost all species. Interestingly, the highest percentage of antioxidant activity (85.7%) was observed in the octaploid *R. persicus*. Total phenolic content improved in *R. persicus* but decreased in *R. caesius* and *R. sanctus*. In *R. caesius*, diploid fruits had the highest content of vitamin C (9.2 mg l^−1^), antioxidants (74.7%), and phenol (236 mg GAE l^−1^). In *Rubus sanctus*, the highest amounts of antioxidants (66.8%) and phenol (233.3 mg GAE l^−1^) were found in diploids, while the highest anthocyanin content (62.2 mg L^−1^) occurred in tetraploids.

The size of conical cells increased by 32.3% in tetraploid plants and by 48.2% in octaploid plants, reaching an average size of 46 μm, as shown by micromorphological changes in the petals of *R. persicus* (Figure 3).

### Nectar sugars

**Figure 4:**
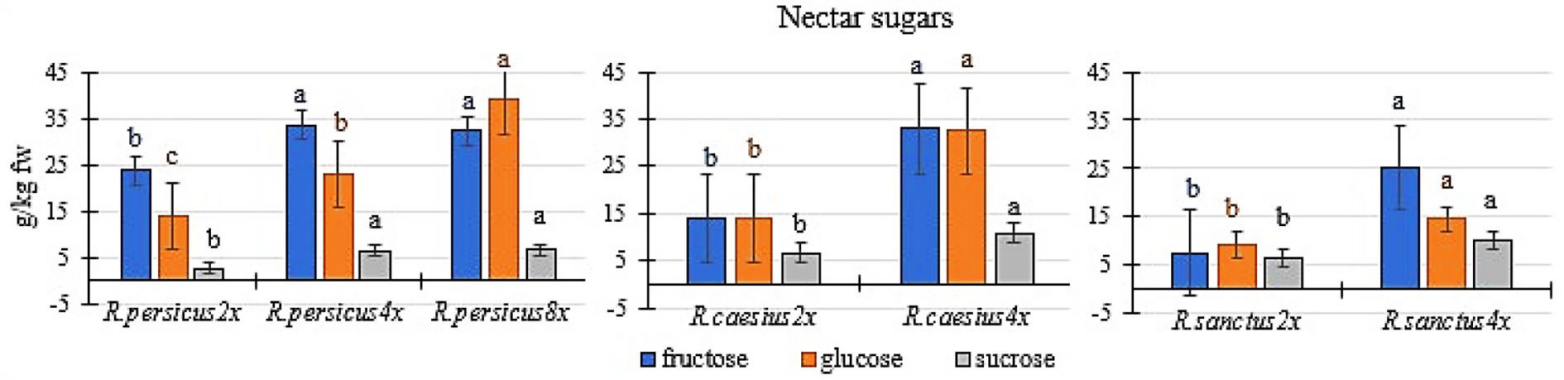
The concentration of fructose, glucose, and sucrose in the nectar of di and polyploidy plant of *R. persicus, R. caesius*, and *R. sanctus*. In each species, means followed by the same letters are not significantly different according to the LSD test at *P* ≤ 0.05 and the columns with no letters were not significant.

As a general trend, polyploidy increased the fructose, glucose, and sucrose content in the nectar of all three species, compared to the diploid mother plants (Figure 4). The flowers of octaploid and tetraploid *R. persicus* and tetraploid *R. caesius* had the highest sugar content in their nectar. *R. persicus 8x* contained 32.30, 38.87, and 6.53 g kg^−1^ FW ^−1^ of fructose, glucose, and sucrose, respectively, while *R. caesius 4x* contained 33.00, 32.42, and 10.7 g kg^−1^ FW ^−1^ of fructose, glucose, and sucrose, respectively. The polyploid plants of *R. persicus* and *R. caesius* had the highest fructose content. The lowest amounts of fructose (7.58 g kg^−1^ FW ^−1^) and glucose (9.23 g kg^−1^ FW ^−1^) were found in the diploid *R. sanctus*. The tetraploid cytotype of this species also contained a lower amount of simple sugars in its nectar than the tetraploids of the other two species.

### Phytohormones

**Figure 5:**
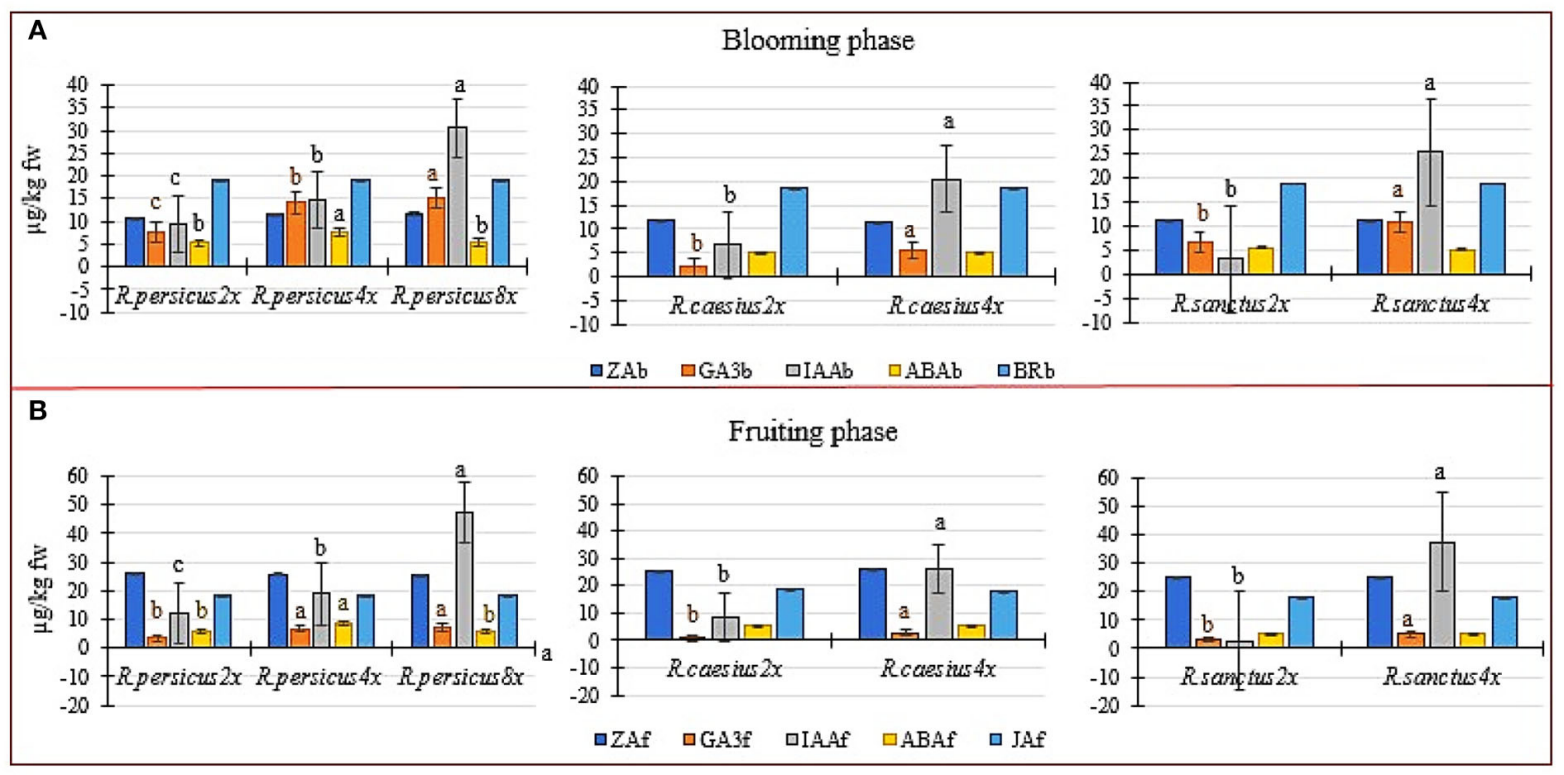
The phytohormones' content in diploid and autopolyploid plants in three blackberry species at the blooming and fruiting phase. **(A)** The concentration of zeatin (ZA), gibberellic acid (GA_3_), indole acetic acid (IAA), abscisic acid (ABA) and brasinoestroid (BR) in the flowering stage of di and polyploid in *R. persicus, R. caesius* and *R. sanctus*. **(B)** the concentration of ZA, GA_3_, IAA, ABA and jasmonic acid (JA) in the fruiting stage of di and polyploid in *R. persicus, R. caesius* and *R. sanctus*. In each species, means followed by the same letters are not significantly different according to the LSD test at *P* ≤ 0.05 and the columns with no letters were not significant.

The polyploid plants of all three species had higher contents of GA_3_ and IAA than their diploid counterparts at both flowering and fruiting stages (Figures 5A,B). However, zeatin, brasinoestroid, ABA (except in the tetraploid *R. persicus*), and jasmonic acid (measured only in the fruiting phase) were not affected by polyploidization in both flowering and fruiting phases in any of the three species (Figures 5A,B). In both flowering and fruiting phases, the maximum and minimum amounts of GA_3_ were measured in *R. persicus 8x* and *R. caesius 2x*, respectively. The octaploid *R. persicus* also had a significantly higher content of IAA during flowering and fruiting phases (36.604 and 47.2136 μg kg^−1^ FW ^−1^, respectively), although the minimum amount of indoleacetic acid was observed in the diploid mother plant of *R. sanctus* during the flowering and fruiting phases (3.18 and 2.81 μg kg^−1^ FW ^−1^, respectively). Induced tetraploid plants of *R. persicus* exhibited significantly higher amounts of abscisic acid than their di- and octaploid cytotypes. Accordingly, the upper limit of ABA was found to be 8.60 μg kg^−1^ FW ^−1^ during fruiting in *R. persicus 4x*.

At different stages of the plant life cycle, the ratio of plant growth regulators is an important factor. Therefore, the ratio of the measured phytohormones was calculated and presented in Table 3. In both flowering and fruiting stages, the ratios of GA_3_/ZA, GA_3_/ABA, IAA/ZA, IAA/ZA, and IAA/ABA increased in the three species due to induced polyploidy, while the general trend for the ratios of GA_3_/IAA, BR/GA_3_, and BR/IAA decreased in all three species. ABA/Zn and BR/ABA did not show a regular pattern, and BR/ZA was not affected by induced polyploidy. During the flowering phase, the highest ratios of GA_3_/ZA, IAA/ZA, and IAA/ABA (1.42, 3.45, and 6.91, respectively) and the lowest ratios of BR/GA_3_ (1.26) and BR/IAA (0.52) were observed in octaploid *R. persicus*. The diploid *R. caesius* exhibited the highest ratio of BR/GA_3_ (10.92), while the highest BR/IAA ratio (8.76) was recorded in *R. caesius 4x*. Meanwhile, *R. sanctus 2x* showed the highest GA_3_/IAA ratio (3.08) and the lowest ratios of IAA/ZA (0.2) and IAA/ABA (0.4), while the tetraploid plants of the same species showed a high ratio of GA_3_/ZA (1.03), GA_3_/ABA (2.12), IAA/ZA (2.74), and IAA/ABA (5.69).

**Table 3:** The mean comparison of phytohormones ratio in diploid and polyploid plants of three blackberry species at flowering and fruiting phase.

**Phase**	**Plants**	**GA_3_/ZA**	**GA_3_/IAA**	**GA_3_/ABA**	**IAA/ZA**	**IAA/ABA**	**ABA/ZA**	**BR/ZA**	**BR/GA_3_**	**BR/IAA**	**BR/ABA**
Blooming phase	*R. persicus 2x*	0.71*^c^	0.81^a^	1.44^c^	0.88^c^	1.79^b^	0.49^b^	1.81	2.53^a^	2.07^a^	3.64^a^
	*R. persicus 4x*	1.29^b^	0.93^a^	1.82^b^	1.38^b^	1.94^b^	0.49^b^	1.81	1.40^b^	1.31^b^	2.55^b^
	*R. persicus 8x*	1.42^a^	0.41^b^	2.86^a^	3.45^a^	6.91^a^	0.71^a^	1.80	1.26^b^	0.52^c^	3.61^a^
		***	***	***	***	***	***	ns	***	***	***
	*R. caesius 2x*	0.16^b^	0.25	0.32^b^	0.62^b^	1.25^b^	0.49	1.75	10.92^a^	2.82^a^	3.55
	*R. caesius 4x*	0.55^a^	0.29	1.15^a^	1.92^a^	3.98^a^	0.48	1.75	3.16^b^	0.91^b^	3.64
		***	ns	***	***	***	ns	ns	***	***	ns
	*R. sanctus 2x*	0.62^b^	3.08^a^	1.29^b^	0.20^b^	0.42^b^	0.48	1.78	2.84^a^	8.76^a^	3.68
	*R. sanctus 4x*	1.025^a^	0.37^b^	2.12^a^	2.74^a^	5.69^a^	0.48	1.78	1.74^b^	0.65^b^	3.69
		***	***	***	***	***	ns	ns	***	***	ns
		GA_3_/ZA	GA_3_/IAA	GA_3_/ABA	IAA/ZA	IAA/ABA	ABA/ZA	JA/ZA	JA/GA_3_	JA/IAA	JA/ABA
Fruiting phase	*R. persicus 2x*	0.13^c^	0.30^a^	0.63^c^	0.46^c^	2.012^b^	0.22^b^	0.71	5.08^a^	1.52^a^	3.21^a^
	*R. persicus 4x*	0.25^b^	0.34^a^	0.76^b^	0.73^b^	2.19 b	0.33^a^	0.70	2.77^b^	0.95^b^	2.10^b^
	*R. persicus 8x*	0.28^a^	0.15^b^	1.25^a^	1.85^a^	8.18^a^	0.22^b^	0.72	2.77^b^	0.38^c^	3.15^a^
		***	***	***	ns	***	***	ns	***	***	***
	*R. caesius 2x*	0.03^b^	0.09	0.14^b^	0.33^b^	1.49^b^	0.22	0.72	22.63^a^	2.15^a^	3.21
	*R. caesius 4x*	0.10^a^	0.10	0.50^a^	1.02^a^	4.74^a^	0.21	0.71	6.51^b^	0.69^b^	3.29
		***	ns	***	***	***	ns	ns	***	***	ns
	*R. sanctus 2x*	0.12^b^	1.13^a^	0.56^b^	0.11^b^	0.50^b^	0.22	0.72	5.79^a^	6.54^a^	3.30
	*R. sanctus 4x*	0.20^a^	0.13^b^	0.93^a^	1.48^a^	6.77^a^	0.21	0.72	3.55^b^	0.49^b^	3.30
		**	***	***	***	***	ns	ns	**	***	ns

^*^Means followed by the same letters in each species are not significantly different according to LSD test.

The “^***^” was significant at P ≤ 0.001, the “^**^” was significant at P ≤ 0.01 and the “ns” letters were not significant.

During the fruiting phase, diploid plants showed higher JA/GA_3_ and JA/IAA ratios than their polyploid counterparts in all three species. Interestingly, the highest ratios of GA_3_/ZA (0.28), GA_3_/ABA (1.25), IAA/ZA (1.85), and IAA/ABA (8.18) were observed in octaploid *R. persicus* during fruiting. The tetraploid cytotype of *R. persicus* had the highest rates of GA_3_/IAA (0.34) and ABA/ZA (0.33). Meanwhile, *R. caesius 2x* had the highest ratio of JA/GA_3_ (22.63), while the highest ratio of JA/IAA (6.54) was measured in *R. sanctus 2x*.

### Correlation and bi-plot analysis

**Figure 6:**
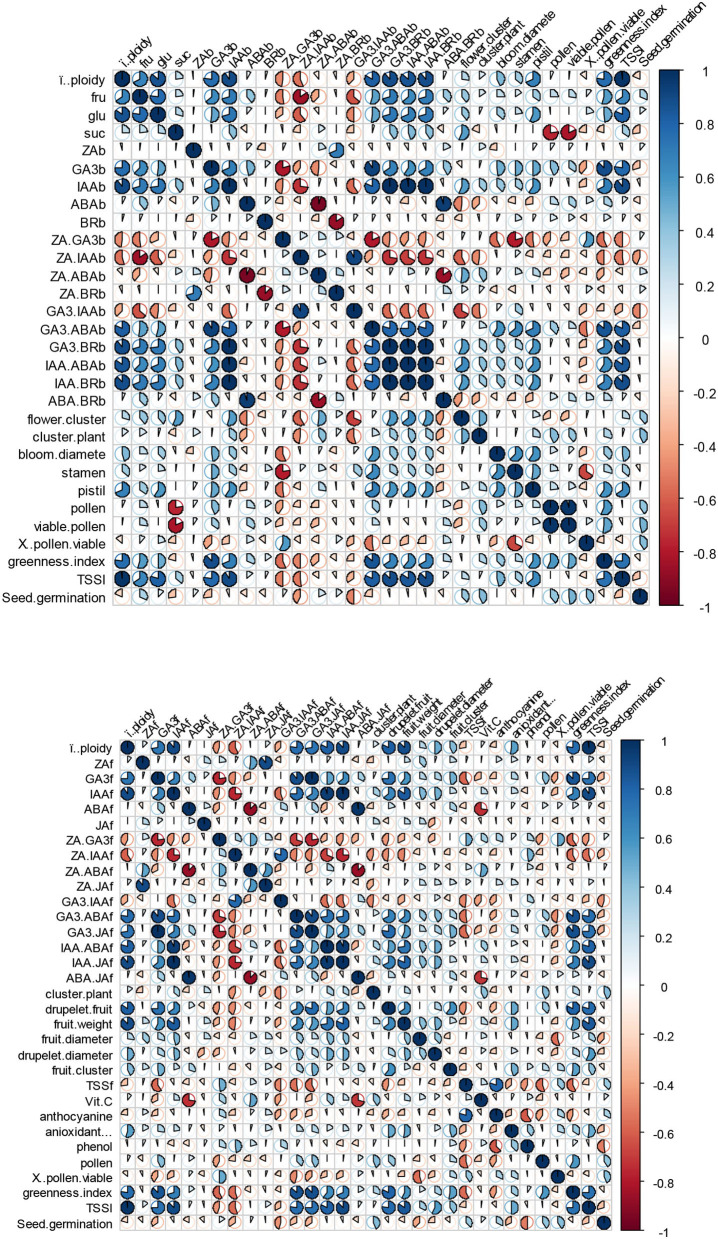
Heat map for the comparison of the recorded traits during blooming (top) and fruiting (down) phases. Each cell is a Pearson's correlation for one feature with the other. Values are reported as −1 to 1 interval. Colors and the pie size are coded by normalized value gradients.

To illustrate the relationships between ploidy level and scored plant attributes as well as the relationships among measured traits, heat maps were generated based on Pearson's correlation. The heat maps were generated separately for the blooming and fruiting phases (Figure 6). The results showed that pistil count, leaf greenness index, leaf TSS, glucose content of flower nectar, IAA and GA3 content, as well as IAA/ABA, IAA/BR, GA_3_/BR, and GA_3_/ABA ratios were strongly and positively associated with ploidy level in the blooming phase (Figure 6, top). Also, the ploidy level correlated significantly and positively with IAA and GA_3_ contents, IAA/ABA, IAA/JA, GA_3_/ABA ratios, fruit weight, drupelet count, leaf TSS, and leaf greenness index during the fruiting stage (Figure 6, down). During both the blooming and fruiting stages, the ZA/IAA ratio correlated negatively with the ploidy level (Figure 6). Many interesting and valuable relationships were also found among other measured attributes. For example, when leaf TSS increased, there was a subsequent increase in the amounts of glucose in the flower nectar, the IAA and GA_3_ contents during the blooming stage. Also, a higher level of leaf TSS in the fruiting stage caused higher concentrations of IAA and GA_3_, drupelet count, fruit weight, and leaf greenness index. For more details, see Figure 6.

**Figure 7:**
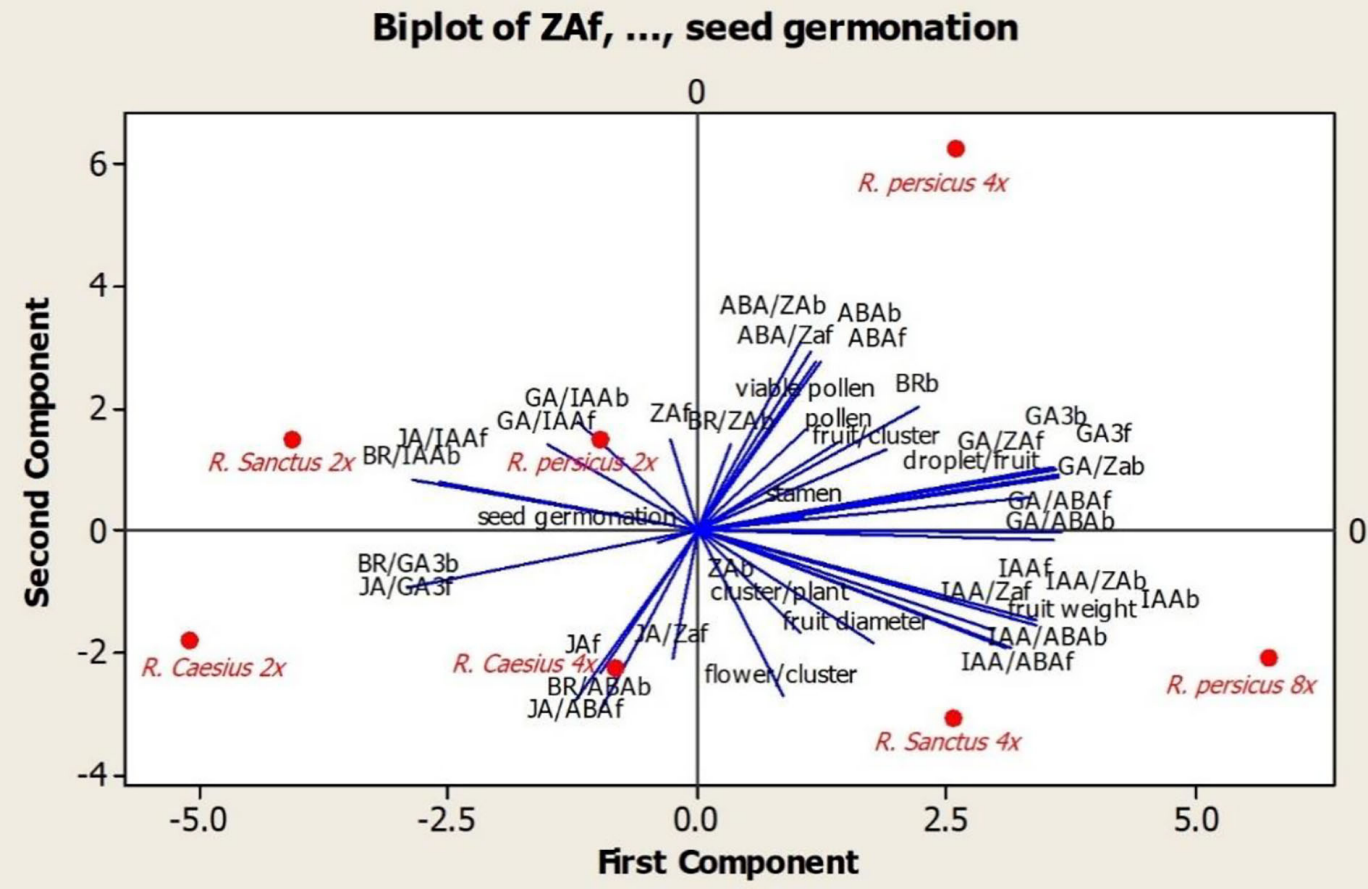
Bi-plot generated based on first two components of the principal components analysis using all measured characteristics of studied *Rubus* species.

To illustrate the relationships of the blackberry genotypes and to determine the influential traits in this grouping, principal component analysis was carried out, and the bi-plot was based on the first two components (Figure 7). The results showed that diploid and tetraploid *R. caesius* plants were separated from other plants and placed in the same quadrant. Plants in this quadrant were characterized by high BR/GA_3_b, BR/ABAb, JA/GA_3_f, JA/ABAf, and JA/ZAf ratios as well as high amounts of JAf. *R. persicus*, and *R. sanctus* were placed in the same quadrant because they had high seed germination rates, a high ZA during the fruiting period, and high ratios of GA_3_/IAAb, GA_3_/IAAf, JA/IAAf, and BR/IAAb. The tetraploid *R. persicus* was placed alone in one quadrant, having high values of reproductive traits (pollen count, viable pollen count, number of stamen and flower/cluster, number of fruit/cluster, number of drupelet/fruit, fruit weight, fruit diameter, and number of cluster/plant) and hormonal quantity (ABAb, ABAf, GAb and GA_3_f, ABA/ZAb, ABA/ZAb, GA_3_/ZAb, GA_3_/ZAf, GA_3_/ABAb, GA_3_/ABAf and BR/ZAb). *R. sanctus 4x* and *R. persicus 8x* were assigned to the last quadrant, which was distinguished by a high number of clusters/plant, flower/cluster, higher fruit weight and size, as well as higher amounts of IAAb, IAAf, and ZAb, and high ratios of IAA/ZAb, IAA/ZAf, IAA/ABAb, and IAA/ABAf.

## Discussion

Polyploidy has occurred frequently in the evolutionary process of many plants (Guo et al., 2017; Peer et al., 2017). As a result of this evolutionary achievement, the pool of genetic variations accessible in polyploids has increased (Baduel et al., 2018). Polyploidy may generate unique plant features, allowing for the development of novel cultivars demanded in the fruit industry. However, various species or cultivars may respond differently to polyploidization, which could be attributed to their origin and genetic structure (Khosravi et al., 2008; Münzbergová, 2017; Regalado et al., 2017). According to the results, the duration of flower bud and fruit growth and development in the octaploid *R. persicus* was much shorter than those of its diploid and tetraploid counterparts, and even compared with other species evaluated herein, which is very important for an early harvest. Changes in the flowering time and fruit development period were reported as a result of polyploidization (Soltis et al., 2004; Dhooghe et al., 2011; Rezende et al., 2020).

Multiplying the number of chromosomes in diploid blackberries not only increased flower and fruit size but also affected flower and fruit morphology. According to the results, the flower, pollen, and fruit sizes of polyploid blackberries were substantially larger than those of diploid plants, with octoploid plants having the largest flower, fruit, and pollen size compared to the diploid and tetraploid plants. Similar results were reported in the case of kiwifruit (Liu et al., 2010; Wu et al., 2012), basil (Omidbaigi et al., 2010), lily (Wu et al., 2007), and muskmelon (Zhang et al., 2010). Cell size and ploidy are frequently linked, and organ size is associated with the degree of polyploidy and cell size (Cheniclet et al., 2005). In this regard, polyploidy induction can generate plants with a more compact stature in some *Rubus* species (Sabooni et al., 2022), facilitating their farm maintenance without the use of hormonal therapies or a labor force to prune blackberry bushes. According to the results, polyploid blackberries performed better in terms of flower/cluster and drupelet/fruit, unlike the tetraploid *Jatropha curcas*, which had a considerably reduced number of flowers and fruits per inflorescence, as well as fewer seeds per fruit when compared to the diploid (Niu et al., 2016). This might be partly attributed to an enhanced pollen fertility in the tetraploid plants or because of enhanced photosynthetic assimilation (Sabooni et al., 2022).

The greenness index and leaf total soluble solids are important factors associated with the photosynthesis rate of leaves. This may influence blackberry yield production (Thompson et al., 2007). A high greenness index can lead to remarkable increases in the amounts of growth-stimulating phytohormones and, subsequently, may boost the photosynthesis rate. This resulted in an enhanced availability of soluble sugars in the leaves and, consequently, a boost in fructose and glucose contents in nectar. Since these were important factors in fertilization, they may have contributed to an increase in yield. These findings are in agreement with those described by Aldesuquy (2015), in which the pretreatment of wheat grain with GA_3_, IAA, or ABA induced a marked increase in leaf pigment and sucrose content, as well as increases in polysaccharides and protein content in the leaves.

The extent and rate of carbon assimilation in the closest leaf to the flower or fruit cluster are very important in blackberries as they can be causes of restriction in total yield (Thompson et al., 2007). Flower development, fruit set, and seed set are all carbon-dependent processes in plants. To set fruits and seeds, flowers need photo-assimilates less than the amount in the succeeding phase of fruit filling and seed maturation (Ruan et al., 2012). There was a clear genetic effect on the flower and fruit attributes, although the ploidy level may possibly account for some of these variations. It has been reported that the relative contents of fruit and seed metabolites in diploid and polyploid plants are considerably different. In this regard, Wang et al. (2022) found that the amounts of lipids, amino acids, their derivatives, and phenolic acids increased significantly in tetraploid plants of both *Oryza indica* and *O. japonica*. Polyploidization frequently leads to gigantism, with an enhanced amount of biomass output and changes in nutritional quality, more carbohydrates, proteins, vitamins, and alkaloids.

To the best of our knowledge, the available literature lacks information on the effect of polyploidization on blackberry nectar quantity and quality. Using high-performance liquid chromatography, we compared the simple sugar composition in the nectars of diploid and polyploid cytotypes of *Rubus* species. The results showed that nectar composition was deeply influenced as the ploidy level increased, which is very important for the attractiveness of flowers to pollinators. The features of the reproductive organs, including inflorescence size, floral display, color, scent chemistry, and nectar quantity, can affect pollinator behavior and pollination efficiency (Husband and Schemske, 2000; Anssour et al., 2009; Balao et al., 2011). In line with the current results, both floral fragrance cues and petal reflectance changed across cytotypes of diploid and tetraploid flowers in *Chamerion angustifolium* (Palmqvist et al., 2021). Also, Davis et al. (1994) reported that haploids of *Brassica rapa* yielded nectar carbohydrates three times less than those of its 2n and 4n lines, which is in general agreement with the findings of the current study. They also discovered that the lateral (inner) pair of glands produced 95 percent of the total nectar carbohydrate per flower at all ploidy levels. These glands were fed only with phloem, which contains more sugar, while the median (outside) glands, which were poor nectar producers, were seldom supplied with phloem (Davis et al., 1994).

The results confirmed that induced-polyploidy changed the flower size and even the number of stamens. It is commonly understood that flower traits are affected by the induction of polyploidy (Balao et al., 2011; Alexander, 2020) which often leads to a shift in pollinator preferences (Muchhala and Potts, 2007; Waterman et al., 2011). For example, polyploidization may lead to an increase in organ size, which could affect floral traits and attractiveness for pollinators. These floral traits include corolla width, flower-pollinator interactions during nectar foraging, corolla tube length, width (Balao et al., 2011), and pollen transfer. Despite their lower frequency in a population, shifts in floral traits between diploids and polyploids may play a significant role in promoting more successful fertilizations within the same cytotype, perhaps leading to preferential pollen transfer between individuals of the minority cytotype (Husband and Sabara, 2004; Kennedy et al., 2006).

Conical cell size is an important factor in flower color and light reflectance. It is directly related to flower attractiveness for pollinators. Any change in this factor can affect the survival of a plant species in its natural habitat (Marques et al., 2016). The size of conical cells of petals was significantly increased in this study, which is consistent with previous results on *Impatiens walleriana* (Ghanbari et al., 2019). Conical epidermal cells have reportedly enhanced floral pigments and color intensity, as they produce a glossy appearance, affect overall petal shape and aroma, reduce wettability, and increase pollinator visits to the flower (Marques et al., 2016).

Polyploid plants had higher levels of growth-stimulating phytohormones. In accordance with the results of this research, Zhang et al. (2020) ascribed the growth advantages of polyploid *Populus* plants to enhanced biosynthesis rates of cytokinin, auxin, gibberellin, and photosynthetic capacity, which lead to greater carbon fixation and higher sugar/starch contents. Plant growth regulators and their related ratios varied dramatically during the blooming process in this study, revealing intricate hormonal connections. When plants bloom, the amounts of IAA and GA_3_ change significantly. The amounts of IAA and GA_3_ in the fruiting stages, as well as the ploidy level, are all key elements in increasing the fruit weight. In addition, the ratios of GA_3_ to ABA, GA_3_ to JA, IAA to JA, and IAA to ABA, as well as fruit drupelet count, greenness index, and TSSl significantly boosted productivity in the fruiting stage. In accordance with the current results, Pei et al. (2019) reported that the amounts of trans zeatin riboside (TZR) were higher right before floral initiation in both diploid and tetraploid radishes, but then declined at flowering time, although the active form of cytokinin (ZT) remained higher in diploid plants compared to the tetraploid ones, supporting the influence of induced polyploidy in hormonal activities. The said research also indicated that, at bolting time, the GA levels in diploid plants were substantially greater than those in tetraploid plants. This was in contrast to the current results, showing that higher GA levels were associated with early bolting phenotypes in diploid plants only, i.e., not in tetraploid plants (Pei et al., 2019). Given the positive correlation between growth-promoting phytohormones, the greenness index, and TSS of leaves, it is reasonable to conclude that an enhanced biosynthesis of phytohormones contributed to larger values of greenness index and TSS, which are both associated with carbon fixation through photosynthesis. Differences among plants in terms of blooming are known to emanate from a range of effective plant growth regulators, e.g., gibberellic acid, abscisic acid, zeatin riboside, indole acetic acid, ethylene, brasinoestroids, jasmonic acid, and strigolactones (Guiqun and Lihua, 2006). Brassinosteroids are phytohormones that regulate cell division and elongation, vascular differentiation, blooming, pollen formation, and photomorphogenesis. Jasmonic acid is largely effective in root growth, seed germination, stamen development, fruit and seed development, and senescence (Šimura et al., 2018; Baghel et al., 2019; Fuentes et al., 2019; Yang et al., 2019). Regarding strawberry fruits, Garrido-Bigotes et al. (2018) revealed a coordinated down-regulation of endogenous jasmonic acid and its biosynthetic genes, occurring from blooming to the ripening phase. Also, Böttcher et al. (2014) reported higher levels of JA in early-stage grape berries, followed by a sharp decrease as the berries matured. The amounts of GA_3_ and IAA were negatively linked with ABA, which is an effective plant growth regulator in fruit and seed maturity. An excess biosynthesis of ABA may deprive the same precursor pool which is necessary for the chlorophyll biosynthesis pathway. A large amount of ABA is generated by the plant itself, thereby promoting seed maturity and causing the plant to enter aging or dormancy (Soto et al., 2013; Bernales et al., 2019; Travisany et al., 2019).

Based on the results of the current study, BR/GA_3_ and BR/ABA ratios were higher in plants during flowering, while JA/ABA, JA/IAA, and JA/GA_3_ were higher than other hormone ratios during fruiting. Flower bud differentiation is promoted by high ratios of ABA/GA_3_, ABA/IAA, ZA/GA_3_, and ZA/IAA. In this regard, a high ZA/GA_3_ ratio was reportedly favorable for apple bud development (Cao et al., 2000), while a high ABA/GA_3_ ratio was helpful for flower bud initiation and differentiation in *Lycoris radiata*. High ZA/IAA and GA_3_/IAA ratios in cotton resulted in a large number of flower buds (Fang et al., 2018). Seed filling in maize and wheat was reportedly regulated by ABA, which promoted embryo development (Yin et al., 2015). In a relevant study, GA_3_/ABA and IAA/ABA ratios increased in the leaves of *R. idaeus* cv. “Heritage” throughout the fruit harvesting period, but then decreased in the last stage. Malik and Archbold (1992) reported that GA_3_/IAA ratios did not vary in the early stages but increased with the progress in blackberry fruit maturity. In addition, a positive relationship was found between GA_3_ and IAA contents. GA_3_ and IAA contents peaked during the rapid expansion of the leaf area, while ABA content peaked during the senescence stage. The number of cotton flower buds increased significantly when the ratio of ZA/IAA and GA_3_/IAA improved (Fang et al., 2018). Interestingly, flower-associated genes responded more strongly to GA_3_ than to benzyl adenine (BA), resulting in variations in flower bud count at the onset of flower bud differentiation (Fang et al., 2018).

Since apomixis can occur in blackberries, the rates of sexual embryonic development and seed germination are influenced by more complicated circumstances. The healthy rate of seed development appears to have been altered in this study due to polyploidization. The diploid plants were affected by ZAf, GA_3_/IAA, JA/IAAf, and BR/IAAb, while polyploidy may have reduced the seed germination rate in blackberry species. The results of correlation analysis showed that seed germination was related to the values of ZAf, JAf, BR/ABAb, BR/IAAb, BR/GA_3_b, JA/ABAf, and JA/GA_3_f. However, unlike fruits, which can develop without pollination, seed development is usually more dependent on effective pollination. Auxins, cytokinin, and gibberellins all play a role in seed development. Problems with auxin production can result in an aberrant embryo morphology, while mutations in the cytokinin pathway may result in larger seeds. Similarly, alterations in gibberellic acid response genes can lead to larger seeds (Ruan et al., 2012). The major stimulators of fruit formation after fertilization are auxin and gibberellin. Higher levels of auxin and gibberellins, as a result of pollination, usually trigger fruit development through cell division and expansion. Auxin can either directly trigger fruit set or indirectly do so by increasing the biosynthesis of GA. This is because auxin, as a treatment, usually leads to a large number of pericarp cells, while GA treatment leads to fewer but larger pericarp cells (Ruan et al., 2012; Lymperopoulos et al., 2018).

## Conclusion

Polyploidy, or whole genome duplication, is a biological phenomenon that has occurred in the plant kingdom throughout evolutionary eras and has improved morpho-physiological features in plants, while also benefiting their adaptability to adverse environmental conditions. For the first time, we investigated the reproductive life cycles of induced autopolyploid and diploid blackberries as an important fruit crop. Overall, the induced polyploidy allowed the *Rubus* species to develop beneficial and unique plant features such as enhanced leaf greenness index, a higher number of flowers or fruit per cluster, larger flowers and berries, higher amounts of nectar sugar, as well as altered levels of phytohormones. Various species responded differently to polyploidization, which could be attributed to their origin and genetic structure. Polyploidy reduced the total number of pollen, the number of viable pollen, and the rate of seed germination. Changes in nectar, flower petal structure, and color can be ascribed to changes that occurred in the size of conical cells on the epidermal surface by induced-polyploidy. According to the results, induced-polyploidy has the potential to be used as an effective breeding tool for breeders who aim at developing sound commercial cultivars. It is recommended that further tests be conducted on their performance in field conditions. These plants can also be used for learning more about the reproductive phase in blackberry and about the mechanisms and gene networks that are responsible for positive changes caused by induced-polyploidy.

## Data availability statement

The raw data supporting the conclusions of this article will be made available by the authors, without undue reservation.

## Author contributions

NS wrote the manuscript, performed the statistical analysis, and conducted all the experiments. AG designed all experiments, provided materials, and supervised the practical issues of the experiments, dealt with data collection, data analysis, and finally reviewed the manuscript. All authors contributed to the article and approved the submitted version.

## Funding

Shiraz University and Iran national Science Foundation (INSF) supported this research financially.

## Conflict of interest

The authors declare that the research was conducted in the absence of any commercial or financial relationships that could be construed as a potential conflict of interest.

## Publisher's note

All claims expressed in this article are solely those of the authors and do not necessarily represent those of their affiliated organizations, or those of the publisher, the editors and the reviewers. Any product that may be evaluated in this article, or claim that may be made by its manufacturer, is not guaranteed or endorsed by the publisher.

## Abbreviations

IAA: indole acetic acid
ZA: zeatin
GA_3_: gibberellic acid
ABA: abscisic acid
JA: jasmonic acid
BR: brasinoestroid
glu: glucose
fru: fructose
suc: sucrose
TSS: total soluble solids.
